# Angiotensin-Converting Enzyme Inhibitors and Angiotensin Receptor Blockers in Patients Undergoing Aortic Valve Replacement for Severe Aortic Stenosis: A Retrospective Cohort Study

**DOI:** 10.3390/jcm15082904

**Published:** 2026-04-10

**Authors:** Husnain Abid, Yusuf Khan, Nazish Khan, Jawad Khan, Richard Paul Steeds

**Affiliations:** 1 Midland Metropolitan University Hospital, Sandwell and West Birmingham NHS Trust, Birmingham B66 2QT, UK; yusuf.khan8@nhs.net (Y.K.); jawad.khan1@nhs.net (J.K.); 2 Queen Elizabeth Hospital, University Hospitals Birmingham NHS Foundation Trust, University of Birmingham, Birmingham B15 2TH, UK; nazish.khan@nhs.net (N.K.); rick.steeds@uhb.nhs.uk (R.P.S.)

**Keywords:** aortic stenosis, ACE inhibitors, angiotensin receptor blockers, TAVI, mortality, renin–angiotensin system

## Abstract

**Background:** Angiotensin-converting enzyme inhibitors (ACEis) and angiotensin receptor blockers (ARBs) were historically considered contraindicated in severe aortic stenosis (AS) due to theoretical haemodynamic risks. Contemporary evidence increasingly challenges this paradigm, yet data on preoperative use and postoperative outcomes remain limited. We examined the association between preoperative ACEi/ARB use and mortality following aortic valve replacement. **Methods:** We conducted a retrospective cohort study of 198 consecutive patients undergoing transcatheter (TAVI) or surgical aortic valve replacement (SAVR) at a single tertiary centre between May 2020 and March 2025. Complete one-year follow up was available for 185 patients (93%). The primary outcome was one-year all-cause mortality. Multivariable logistic regression adjusted for age, sex, hypertension, diabetes, LVEF, and procedure type. **Results:** Of 198 patients, 80 (40%) were receiving ACEi/ARB therapy preoperatively. ACEi/ARB users had a higher prevalence of hypertension (82% vs. 53%, *p* < 0.001) and diabetes (48% vs. 27%, *p* = 0.005) but similar age, valve area, and ejection fraction. Unadjusted one-year mortality was lower in the ACEi/ARB group (7% vs. 19%; odds ratio [OR] 0.33, 95% CI 0.12–0.91, *p* = 0.030). After multivariable adjustment for confounders including age, diabetes, and hypertension, the association did not reach statistical significance (adjusted OR 0.33, 95% CI 0.10–1.12, *p* = 0.075). Among diabetic patients, unadjusted one-year mortality was numerically lower in the ACEi/ARB group (12% vs. 35%, *p* = 0.038); however, six subgroup comparisons were performed and this result would not survive Bonferroni correction (threshold *p* < 0.008). This exploratory finding should be interpreted with caution given the small sample size and absence of adjustment for confounders. **Conclusions:** Preoperative ACEi/ARB use was associated with lower unadjusted one-year mortality, but this association did not reach statistical significance after multivariable adjustment and residual confounding cannot be excluded. ACEi/ARB use was not associated with increased mortality in this cohort. These hypothesis-generating findings from a single-centre observational study require confirmation in adequately powered prospective trials.

## 1. Introduction

Aortic stenosis (AS) is the most prevalent valvular heart disease in the developed world, with an increasing burden driven by population ageing. Once symptoms develop, prognosis deteriorates markedly, and aortic valve replacement—surgical (SAVR) or transcatheter (TAVI)—remains the established intervention for symptomatic disease. For much of the twentieth century and into the early 2000s, angiotensin-converting enzyme inhibitors (ACEis) and angiotensin receptor blockers (ARBs) were considered contraindicated in patients with moderate to severe AS [1,2]. This view was based on classical haemodynamic principles rather than outcome-based clinical evidence.

Severe AS imposes a fixed obstruction to left ventricular outflow, and it was hypothesised that vasodilator therapy could precipitate hypotension, compromise coronary perfusion, and provoke syncope, myocardial ischaemia, or sudden cardiac death [1]. These concerns were reinforced by early invasive haemodynamic studies and anecdotal reports. Consequently, ACEis and ARBs were considered contraindicated or to be used only with caution in severe AS, particularly in symptomatic patients [1,2].

Contemporary understanding recognises that AS is not solely a mechanical disease of valvular obstruction but also a condition characterised by profound myocardial and neurohormonal changes. Chronic pressure overload leads to concentric left ventricular hypertrophy, increased myocardial oxygen demand, reduced capillary density, and progressive interstitial fibrosis [3,4]. Activation of the renin–angiotensin–aldosterone system (RAAS) is a central feature of pressure-overload states, promoting cardiomyocyte hypertrophy, fibroblast activation, and collagen deposition [3,4].

The landmark RIAS trial demonstrated that ramipril was safe and reduced left ventricular mass in asymptomatic moderate to severe AS [5]. Large observational studies, predominantly from cohorts examined after intervention, including analyses from the STS/ACC TVT Registry and PARTNER trials, have shown that RAAS inhibitor use is associated with 18–24% reductions in all-cause mortality [6,7,8]. Meta-analyses pooling over 30,000 TAVR patients report consistent associations with lower mortality without increased adverse events [9].

Despite this accumulating evidence from postintervention cohorts, data on preoperative ACEi/ARB prescribing and its association with postoperative outcomes remain limited. We therefore examined the relationship between preoperative ACEi/ARB use and mortality in a consecutive cohort of patients undergoing aortic valve replacement, hypothesising that RAAS inhibition would be associated with lower mortality.

## 2. Materials and Methods

### 2.1. Study Design and Population

This retrospective cohort study analysed consecutive patients undergoing aortic valve replacement at a single tertiary cardiac centre between May 2020 and March 2025 (no procedures were performed between January and April 2020 owing to suspension of elective cardiac surgery during the initial COVID-19 pandemic wave). Data were extracted from institutional surgical and echocardiographic databases and cross-referenced with electronic medical records and national mortality registries.

**Figure 1:**
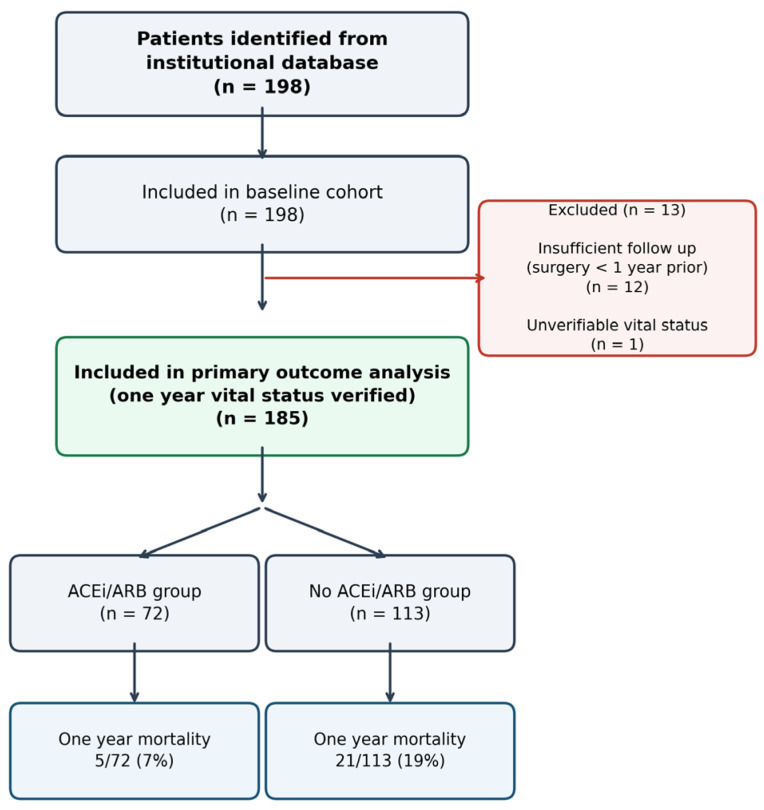
Flow diagram showing study cohort selection from 198 patients identified from the institutional database to 185 included in the primary outcome analysis. Thirteen patients were excluded: 12 with insufficient follow up (surgery < 1 year prior) and one with unverifiable vital status. Among the 185 patients with verified one-year vital status, 72 were receiving preoperative ACEi/ARB therapy and 113 were not.

A total of 198 patients were identified from the institutional database (Figure 1). All 198 had complete demographic, medication, and baseline echocardiographic data. For the primary endpoint analysis, 13 patients were excluded: 12 had undergone their procedure within the preceding 12 months and therefore had insufficient follow up to ascertain one-year mortality status, and one patient’s vital status could not be verified through institutional or national registry records. The final analytical cohort comprised 185 patients with complete one-year outcome data, representing a 93% follow-up rate.

### 2.2. Data Collection and Definitions

Demographic and clinical data included age at surgery, sex, and body mass index (BMI). Documented comorbidities comprised hypertension, diabetes mellitus, atrial fibrillation, and hypercholesterolaemia. Preoperative medications were recorded from preadmission clinic documentation and admission drug charts, including β-blockers, ACEis, ARBs, mineralocorticoid receptor antagonists, SGLT2 inhibitors, and GLP-1 receptor agonists. ACEi/ARB use was defined as a documented prescription of an ACEi or ARB at the most recent preadmission assessment or on the admission drug chart on the day of the procedure. ACEi and ARB use was combined into a single exposure variable owing to the shared pharmacological mechanism of RAAS inhibition and the small number of ARB-only users.

Echocardiographic parameters included absolute aortic valve area (AVA), indexed valve area (AVAi), peak aortic jet velocity, mean transvalvular gradient, left ventricular ejection fraction (LVEF), and left ventricular mass index (LVMi), calculated using the Devereux formula from M-mode measurements where available (*n* = 152). Functional status was assessed using the New York Heart Association (NYHA) classification.

### 2.3. Outcomes

The primary outcome was one-year all-cause mortality. The secondary outcome was overall all-cause mortality, defined as death from any cause occurring at any point during the follow-up period (from date of procedure to the censor date of 24 February 2026). The latest recorded death occurred in November 2025. The censor date of 24 February 2026 was added during the revision process as a formally defined administrative endpoint for the secondary outcome follow-up period. It represents the date on which we completed our final data verification and defined the observational window, not a date on which new data collection or registry linkage was performed. Given that the study period commenced in May 2020, with a median follow up of 2.2 years, the majority of patients did not have sufficient follow-up duration for five-year outcome ascertainment; accordingly, five-year mortality was not analysed. Mortality data were obtained from institutional records and verified against national registries. Data on heart failure hospitalisation were not available from the institutional database and could not be included as an endpoint.

### 2.4. Statistical Analysis

Continuous variables were assessed for normality using the Shapiro–Wilk test. Nonparametric tests were applied due to non-normal distributions. Baseline characteristics were compared using Mann–Whitney U tests for continuous variables and chi-square or Fisher’s exact tests for categorical variables. NYHA functional class was analysed as an ordinal variable using the Mann–Whitney U test.

The association between ACEi/ARB use and mortality was assessed using Fisher’s exact test with calculation of odds ratios and 95% confidence intervals. A multivariable logistic regression model adjusted for clinically relevant confounders including age, sex, hypertension, diabetes, LVEF, and procedure type. Model fit was assessed using the Hosmer–Lemeshow test and Nagelkerke R^2^ statistic.

**Figure 2:**
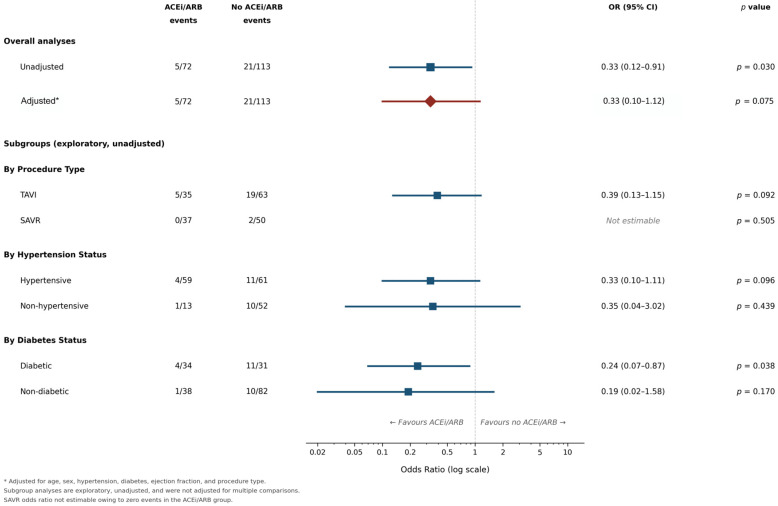
Forest plot showing the overall unadjusted and adjusted odds ratios with 95% confidence intervals for one-year all-cause mortality associated with preoperative ACEi/ARB use. Subgroup odds ratios are displayed for visual reference only; given the sparse data and small sample sizes within the strata, these estimates are unstable and no inference of effect modification should be drawn. Table 4 provides the primary subgroup reporting using event counts, risk differences, and Fisher’s exact test *p* values. Subgroup denominators correspond to Table 4. Squares represent point estimates; the vertical dashed line represents the null effect (OR = 1.0). Dark blue squares represent unadjusted point estimates; the dark red diamond represents the adjusted overall estimate. * Adjusted for age, sex, hypertension, diabetes, LVEF, and procedure type (TAVI vs. SAVR).

Prespecified subgroup analyses examined the association between ACEi/ARB use and mortality stratified by procedure type (TAVI vs. SAVR), hypertension status, and diabetes status. Event rates were compared using Fisher’s exact tests; given the sparse data within strata, subgroup odds ratios with confidence intervals were computed for graphical display only (Figure 2) but are not reported numerically given the instability of estimates from sparse data, and these analyses are considered exploratory. Statistical significance was defined as *p* < 0.05 (two-tailed). Results with *p* values above this threshold are reported as not statistically significant; any observed trends are described for completeness but are considered exploratory and hypothesis-generating only. Analyses used IBM SPSS Statistics (version 31.0.2.0) and Python (version 3.11). Complete demographic, medication, and baseline echocardiographic data were available for all 198 patients with the exception of left ventricular mass index, which was available in 152 patients (77%); this variable was not included in the multivariable model. Operative risk scores such as EuroSCORE II were not recorded in the institutional database and therefore could not be compared between groups. Claude (version 3.5 Sonnet; Anthropic, San Francisco, CA, USA) was used for writing assistance and language editing during the preparation of this manuscript. The AI tool was not used for data analysis, statistical interpretation, or generation of scientific content. All intellectual content, analytical decisions, and conclusions are the sole responsibility of the authors.

### 2.5. Ethical Considerations

This study was conducted in accordance with institutional governance standards for audit and service evaluation. All data were anonymised prior to analysis. Formal research ethics committee approval was not required for this retrospective, noninterventional analysis of routinely collected clinical data.

## 3. Results

### 3.1. Baseline Characteristics

The study included 198 patients (Table 1). Baseline characteristics are reported for the full cohort (*n* = 198), while analyses of one-year mortality are restricted to patients with verified one-year vital status (*n* = 185). Eighty patients (40%) were receiving ACEi/ARB therapy at the time of valve replacement. The ACEi/ARB and non-ACEi/ARB groups were similar in age (median 74 vs. 77 years, *p* = 0.222) and sex distribution (45% vs. 40% female, *p* = 0.564). Patients receiving ACEi/ARB therapy had a higher BMI (median 29.4 vs. 27.4 kg/m^2^, *p* = 0.001) and significantly higher prevalence of hypertension (82% vs. 53%, *p* < 0.001) and diabetes (48% vs. 27%, *p* = 0.005), which are established indications for ACEi/ARB therapy.

Echocardiographic parameters were comparable between groups. Absolute aortic valve area (median 0.80 cm^2^ in both groups, *p* = 0.147), indexed valve area (0.43 vs. 0.41 cm^2^/m^2^, *p* = 0.167), mean gradient (44 vs. 45 mmHg, *p* = 0.662), and LVEF (55% vs. 55%, *p* = 0.591) did not differ significantly. LV mass index, available in 152 patients, was similar between groups (111 vs. 113 g/m^2^, *p* = 0.920). However, ACEi/ARB users presented with more severe functional limitation, with 51% in NYHA class III–IV compared to 33% of nonusers (*p* = 0.002).

Procedure type distribution was similar, with 46% of ACEi/ARB users undergoing TAVI compared to 56% of nonusers (*p* = 0.233). Use of other cardiovascular medications did not differ significantly between groups.

### 3.2. Primary Outcome: One-Year Mortality

Complete one-year follow-up data were available for 185 of 198 patients (93%). Thirteen patients were excluded from the primary analysis: 12 had undergone their procedure within the preceding 12 months and therefore had insufficient follow up, and one patient’s vital status could not be verified.

One-year all-cause mortality was lower in patients receiving ACEi/ARB therapy: 5 of 72 patients (7%) versus 21 of 113 patients (19%) in the non-ACEi/ARB group (Table 2). The unadjusted odds ratio was 0.33 (95% CI 0.12–0.91, *p* = 0.030).

After adjustment for age, sex, hypertension, diabetes, LVEF, and procedure type in multivariable logistic regression (Table 3), the point estimate for ACEi/ARB use was identical to the unadjusted estimate (adjusted OR 0.33, 95% CI 0.10–1.12, *p* = 0.075); however, the widened confidence interval crossed 1 and this result did not reach statistical significance. Residual confounding by unmeasured factors remains possible. With only 26 outcome events across seven covariates (approximately 3.7 events per variable), the model substantially exceeds established limits for reliable logistic regression and coefficient estimates should be interpreted with caution. Diabetes was the strongest independent predictor of one-year mortality (OR 4.93, 95% CI 1.68–14.48, *p* = 0.004), while hypertension was independently associated with lower mortality (OR 0.25, 95% CI 0.08–0.77, *p* = 0.016); the direction of this association is discussed in Section 4. Age was also a significant predictor (OR 1.12 per year, 95% CI 1.02–1.21, *p* = 0.013).

### 3.3. Secondary Outcomes

Overall mortality during the entire follow-up period (median 2.2 years, IQR 1.9–3.7 years) was 14% in the ACEi/ARB group (10 of 72 patients) versus 26% in nonusers (29 of 113 patients), corresponding to an unadjusted OR of 0.47 (95% CI 0.21–1.03, *p* = 0.065). This difference did not reach statistical significance. The attenuation of the apparent association over longer follow up may reflect convergence of mortality risk over time, changes in postoperative management, or the influence of competing risks and non-cardiovascular mortality. As follow-up duration varied between patients (IQR 1.9–3.7 years), this analysis does not account for differential censoring and a time-to-event approach would be more appropriate for this endpoint.

### 3.4. Subgroup Analyses

Exploratory subgroup analyses are presented in Table 4 and should be interpreted with caution given the limited sample sizes and sparse event counts within each stratum. Among diabetic patients, one-year mortality was numerically lower in the ACEi/ARB group (4/34 [12%] vs. 11/31 [35%]; Fisher’s exact test, *p* = 0.038); however, this subgroup comprised only 65 patients and the finding should be regarded as hypothesis-generating. TAVI recipients showed a numerically lower mortality rate with ACEi/ARB use (5/35 [14%] vs. 19/63 [30%], *p* = 0.092), though this did not reach statistical significance. Hypertensive patients showed a similar nonsignificant pattern (4/59 [7%] vs. 11/61 [18%], *p* = 0.096). Among SAVR recipients, there were no events in the ACEi/ARB group (0/37) versus 2/50 (4%) in nonusers (*p* = 0.505), precluding meaningful comparison. None of these subgroup comparisons were adjusted for confounders.

## 4. Discussion

This study found that preoperative ACEi/ARB use was associated with lower unadjusted one-year mortality following aortic valve replacement for severe aortic stenosis (7% vs. 19%, OR 0.33, *p* = 0.030). The adjusted analysis showed an identical point estimate (adjusted OR 0.33) but the wider confidence interval included the null value (95% CI 0.10–1.12, *p* = 0.075), and this result was not statistically significant. Given the limited number of outcome events (26 deaths among 185 patients, approximately 3.7 events per variable across seven covariates), the model substantially exceeds established limits for reliable logistic regression, and the possibility of a chance finding cannot be excluded. The counterintuitive direction of the hypertension coefficient (OR 0.25, *p* = 0.016) likely reflects collinearity with ACEi/ARB prescribing, treatment selection, or residual confounding rather than a causal protective effect of hypertension itself. Among diabetic patients, a nominally significant difference in unadjusted event rates was observed (12% vs. 35%, *p* = 0.038), though this exploratory subgroup analysis requires cautious interpretation given the small sample and absence of adjustment for confounders.

### 4.1. Comparison with the Existing Literature

Our findings are broadly consistent with previous observational evidence, which has predominantly examined postintervention RAAS inhibitor use. The Nadir et al. study of 2117 patients with AS demonstrated that ACEi/ARB use was associated with an adjusted hazard ratio of 0.76 for all-cause mortality [6]. The JAMA 2018 analysis by Inohara et al. of 21,312 TAVR patients from the STS/ACC TVT Registry showed a one-year mortality of 12.5% in patients receiving RAAS inhibitors versus 14.9% in nonusers (HR 0.82, 95% CI 0.76–0.90) [7]. The PARTNER 2 secondary analysis by Chen et al. reported a two-year all-cause mortality of 18.6% in patients receiving RAAS inhibitors versus 27.5% in nonusers (*p* < 0.0001) [8].

This distinction between preoperative and postoperative prescribing is clinically important. Patients established on RAAS inhibitors before intervention may tolerate therapy better and may represent a selected population with favourable medication adherence and healthcare engagement. Whether longer preoperative exposure to RAAS inhibition confers additional myocardial remodelling benefit is speculative and cannot be determined from these data. To our knowledge, few studies have examined preoperative prescribing as the primary exposure of interest.

### 4.2. Mechanistic Considerations

The proposed effects of RAAS inhibition in AS may extend beyond blood pressure reduction. The Friedrich et al. study using intracoronary enalaprilat demonstrated that local ACE inhibition improved diastolic function in AS patients with LV hypertrophy, independent of systemic effects [10]. O’Brien et al. showed that ACE and angiotensin II are present within stenotic aortic valve leaflets themselves, suggesting local RAAS activation may contribute to disease pathophysiology [11].

The RIAS trial demonstrated that ramipril reduced LV mass in asymptomatic AS patients (−3.9 g vs. +4.5 g, *p* = 0.006), suggesting that beneficial remodelling may occur despite fixed valvular obstruction [5]. In our cohort, LV mass index did not differ between groups (111 vs. 113 g/m^2^, *p* = 0.920), though this cross-sectional comparison cannot establish whether RAAS inhibition modifies myocardial remodelling in this context—potentially including reduction in myocardial fibrosis, improvement in diastolic function, and attenuation of neurohormonal activation, none of which were assessed in the present study.

The numerically lower mortality in TAVI recipients (14% vs. 30%, *p* = 0.092) did not reach statistical significance, and given the observational design, mechanistic inferences should not be drawn. TAVI recipients are typically older with greater comorbidity burden, and any apparent difference may reflect confounding by indication or unmeasured differences between groups rather than a true treatment effect.

### 4.3. Clinical Implications

These findings have potential clinical relevance but must be interpreted within the limitations of a single-centre retrospective study with a nonsignificant adjusted primary analysis. Current guidelines support the use of ACEis and ARBs in patients with severe AS who have standard indications such as hypertension, diabetes with proteinuria, or heart failure; this recommendation is based on existing guideline evidence rather than the present study. Our data did not demonstrate increased mortality associated with preoperative RAAS inhibition; however, the observational design and nonsignificant adjusted analysis preclude definitive conclusions about either harm or benefit, and adequately powered prospective studies are needed. The unadjusted absolute risk difference was 12 percentage points (19% minus 7%); however, given that the adjusted analysis did not reach statistical significance, calculation of a number needed to treat is not appropriate and no treatment effect estimate should be drawn from unadjusted figures alone.

Current guidelines have evolved to acknowledge the safety and potential benefit of RAAS inhibitors in AS. The 2021 ESC/EACTS Guidelines state that ACEis are safe in aortic stenosis when blood pressure is monitored carefully and may have beneficial myocardial effects [12]. The 2020 ACC/AHA Guidelines provide a Class I recommendation for treating hypertension in AS patients according to standard guideline-directed therapy and a Class IIb recommendation for RAAS blocker therapy post-TAVI [13]. Our data are compatible with these recommendations, though the nonsignificant adjusted analysis precludes conclusions regarding independent benefit from the present study.

### 4.4. Limitations

Several limitations warrant consideration. The retrospective, single-centre design limits generalisability and precludes causal inference. Selection bias is a significant concern: patients prescribed ACEis/ARBs likely differed from nonusers in ways not fully captured by measured confounders. The higher prevalence of hypertension and diabetes in the ACEi/ARB group suggests appropriate prescribing for these indications, and residual confounding by indication remains possible despite adjustment. Operative risk scores such as EuroSCORE II were not recorded, so residual confounding related to treatment selection (TAVI versus SAVR) and ACEi/ARB prescribing cannot be excluded.

While our population (*n* = 198) is smaller than the major postintervention registries (≥2000 patients), our study addresses a distinct clinical question, preoperative prescribing, not examined in those larger cohorts. The unadjusted analysis reached statistical significance (*p* = 0.030), while the multivariable model did not (*p* = 0.075). With only 26 events across seven covariates (approximately 3.7 events per variable, substantially below the recommended minimum of 10), the model was underpowered and at risk of overfitting, and the nonsignificant adjusted result may reflect insufficient statistical power, residual confounding, or the absence of a true independent effect; these possibilities cannot be distinguished in the present dataset. The nominally significant finding in the diabetic subgroup (*p* = 0.038) should be regarded as hypothesis-generating, as six subgroup comparisons were performed without correction for multiple testing (Bonferroni-adjusted threshold *p* < 0.008), and the small sample size within this stratum limits reliability.

NYHA functional class differed significantly between groups (51% vs. 33% in class III–IV, *p* = 0.002) and is a recognised predictor of postoperative mortality but was not included in the multivariable model because the addition of a further covariate would have exacerbated overfitting (events per variable approximately 3.3). As the ACEi/ARB group had worse functional status, this omission would, if anything, bias the adjusted estimate toward the null rather than inflate an apparent benefit; however, this cannot be confirmed without formal adjustment.

We lacked data on medication doses, adherence, duration of therapy before surgery, and postoperative medication changes. Left ventricular mass was estimated using the Devereux formula from M-mode measurements, which has recognised limitations compared with three-dimensional echocardiography or cardiac magnetic resonance. Data on heart failure hospitalisation and time from diagnosis to intervention were not available from the institutional database. Patients who tolerated ACEis/ARBs preoperatively may represent a healthier population with better blood pressure control and overall cardiovascular status. The relatively advanced age of the study population (group medians of 74 and 77 years) reflects contemporary referral patterns at a tertiary centre performing both SAVR and TAVI during the study period (2020–2025), in which an increasing proportion of patients are elderly and referred for TAVI. Valve type (mechanical versus bioprosthetic) was not available in the dataset and could not be analysed.

The baseline imbalance in diabetes prevalence between groups (48% in the ACEi/ARB group versus 27% in the non-ACEi/ARB group, *p* = 0.005) warrants careful consideration. Diabetes is a well-established independent risk factor for mortality following aortic valve replacement and was the strongest predictor of one-year mortality in our multivariable model (OR 4.93, *p* = 0.004). Although the adjusted analysis accounted for diabetes, residual confounding by diabetes severity, glycaemic control, and associated comorbidities remains possible. The apparently lower unadjusted mortality associated with ACEi/ARB use in diabetic patients may reflect the known renoprotective and cardioprotective effects of RAAS inhibition in this population, or it may represent confounding by indication, as patients with diabetes and proteinuria are more likely to be prescribed ACEi/ARB therapy.

Several features of the results merit further reflection. The primary outcome (unadjusted one-year mortality) reached statistical significance (*p* = 0.030), but the adjusted analysis did not (*p* = 0.075), and the overall mortality comparison over the full follow-up period was also not statistically significant (*p* = 0.065). The attenuation of the apparent association at longer follow up may reflect non-proportional hazards, whereby any early difference in mortality diminishes over time as competing causes of death accumulate. Alternatively, the initial unadjusted finding may represent a chance observation in the context of residual confounding, and the true independent effect of preoperative ACEi/ARB use may be smaller than the point estimate suggests—or absent. Given the limited sample size, the number of statistical comparisons performed, and the observational design with residual confounding, the possibility that the unadjusted primary outcome represents a chance finding cannot be excluded.

### 4.5. Future Directions

Adequately powered, multi-centre prospective studies—ideally randomised controlled trials—are needed to determine whether preoperative RAAS inhibition confers an independent survival benefit following aortic valve replacement. Such studies should incorporate standardised operative risk scoring, detailed medication adherence data, and serial echocardiographic follow up to evaluate myocardial remodelling. Integration of data from national registries with linked prescribing records could provide larger sample sizes and reduce the confounding inherent in single-centre observational designs.

## 5. Conclusions

In this retrospective cohort study, preoperative ACEi/ARB use was associated with lower unadjusted one-year all-cause mortality following aortic valve replacement for severe aortic stenosis (OR 0.33, 95% CI 0.12–0.91, *p* = 0.030). After multivariable adjustment, this association did not reach statistical significance (adjusted OR 0.33, 95% CI 0.10–1.12, *p* = 0.075), and a causal relationship cannot be inferred from these data. Preoperative ACEi/ARB use was not associated with increased mortality in this cohort. These findings are hypothesis-generating and are compatible with current guideline recommendations regarding RAAS inhibitor use in AS, though the nonsignificant adjusted analysis precludes conclusions about independent benefit from the present study. Whether preoperative initiation of ACEi/ARB therapy confers an independent survival benefit remains uncertain; adequately powered prospective randomised trials are needed to address this question.

## Figures and Tables

**Table 1:** Baseline characteristics of study participants by ACEi/ARB status.

Characteristic	ACEi/ARB (*n* = 80)	No ACEi/ARB (*n* = 118)	*p* Value
Demographics			
Age at surgery, years	74 (68–80)	77 (65–82)	0.222
Female sex, *n* (%)	36 (45)	47 (40)	0.564
BMI, kg/m^2^	29.4 (26.5–33.4)	27.4 (25.0–30.0)	0.001
Comorbidities			
Hypertension, *n* (%)	66 (82)	62 (53)	<0.001
Diabetes, *n* (%)	38 (48)	32 (27)	0.005
Atrial fibrillation, *n* (%)	17 (21)	23 (19)	0.903
Echocardiographic Findings			
Valve area, cm^2^	0.80 (0.70–0.90)	0.80 (0.66–0.90)	0.147
Mean gradient, mmHg	44 (38–56)	45 (32–56)	0.662
LVEF, %	55 (54–60)	55 (51–62)	0.591
LV mass index, g/m^2^ *	111 (87–138)	113 (86–140)	0.920
Clinical Presentation			
NYHA III–IV, *n* (%)	41 (51)	39 (33)	0.002
TAVI procedure, *n* (%)	37 (46)	66 (56)	0.233

Values are median (IQR) or *n* (%). * LV mass index calculated using the Devereux formula; available in 152 patients. BMI, body mass index; LVEF, left ventricular ejection fraction; NYHA, New York Heart Association; TAVI, transcatheter aortic valve implantation.

**Table 2:** Mortality outcomes by ACEi/ARB status.

Outcome	ACEi/ARB	No ACEi/ARB	OR (95% CI)	*p* Value
One-year mortality	5/72 (7%)	21/113 (19%)	0.33 (0.12–0.91)	0.030
Overall mortality	10/72 (14%)	29/113 (26%)	0.47 (0.21–1.03)	0.065

Values are *n*/*N* (%). OR, odds ratio; CI, confidence interval. Both outcomes are reported for the 185 patients with verified one-year vital status; the same analytical cohort was used for the secondary outcome for consistency.

**Table 3:** Multivariable logistic regression for one-year mortality.

Variable	Adjusted OR	95% CI	*p* Value
ACEi/ARB use	0.33	0.10–1.12	0.075
Age (per year)	1.12	1.02–1.21	0.013
Female sex	0.83	0.29–2.42	0.738
Hypertension	0.25	0.08–0.77	0.016
Diabetes	4.93	1.68–14.48	0.004
LVEF (per %)	0.97	0.93–1.01	0.174
TAVI procedure	4.86	0.81–29.30	0.084

Nagelkerke R^2^ = 0.386; Hosmer–Lemeshow goodness of fit χ^2^ = 6.78, df = 8, *p* = 0.560. Model based on 185 patients with 26 events (3.7 events per variable). Individual covariate odds ratios should be interpreted with caution given the low events-per-variable ratio; wide confidence intervals for several covariates reflect model instability. LVEF, left ventricular ejection fraction; OR, odds ratio; CI, confidence interval; TAVI, transcatheter aortic valve implantation.

**Table 4:** Exploratory subgroup analyses: unadjusted one-year mortality by ACEi/ARB status.

Subgroup	ACEi/ARB	No ACEi/ARB	Risk Difference	*p* Value
By Procedure Type				
TAVI	5/35 (14%)	19/63 (30%)	−16%	0.092
SAVR	0/37 (0%)	2/50 (4%)	−4%	0.505
By Hypertension Status				
Hypertensive	4/59 (7%)	11/61 (18%)	−11%	0.096
Non-hypertensive	1/13 (8%)	10/52 (19%)	−11%	0.439
By Diabetes Status				
Diabetic	4/34 (12%)	11/31 (35%)	−24%	0.038
Non-diabetic	1/38 (3%)	10/82 (12%)	−9%	0.170

Values are *n*/*N* (%). Risk difference is the unadjusted absolute difference in event rates (ACEi/ARB minus no ACEi/ARB). SAVR, surgical aortic valve replacement; TAVI, transcatheter aortic valve implantation. *p* values from Fisher’s exact tests. All subgroup analyses are exploratory, were not adjusted for confounders or multiple comparisons, and should be interpreted with caution given the sparse data. Six subgroup comparisons were performed; the diabetic subgroup result (*p* = 0.038) would not survive correction for multiple testing (Bonferroni-adjusted threshold *p* < 0.008).

## Data Availability

The data presented in this study are available on request from the corresponding author. The data are not publicly available due to patient confidentiality requirements.

## Abbreviations

The following abbreviations are used in this manuscript:

ACEi	Angiotensin-converting enzyme inhibitor
ARB	Angiotensin receptor blocker
AS	Aortic stenosis
AVA	Aortic valve area
AVAi	Indexed aortic valve area
BMI	Body mass index
CI	Confidence interval
LVEF	Left ventricular ejection fraction
LVMi	Left ventricular mass index
NYHA	New York Heart Association
OR	Odds ratio
RAAS	Renin–angiotensin–aldosterone system
SAVR	Surgical aortic valve replacement
TAVI	Transcatheter aortic valve implantation
